# A general scenario of *Hox *gene inventory variation among major sarcopterygian lineages

**DOI:** 10.1186/1471-2148-11-25

**Published:** 2011-01-26

**Authors:** Dan Liang, Riga Wu, Jie Geng, Chaolin Wang, Peng Zhang

**Affiliations:** 1 State Key Laboratory of Biocontrol, Key Laboratory of Gene Engineering of the Ministry of Education, School of Life Sciences, Sun Yat-Sen University, Guangzhou 510275, China; 2 Alligator Research Center of Anhui Province, Xuanzhou 242000, Anhui, China

## Abstract

**Background:**

*H*ox genes are known to play a key role in shaping the body plan of metazoans. Evolutionary dynamics of these genes is therefore essential in explaining patterns of evolutionary diversity. Among extant sarcopterygians comprising both lobe-finned fishes and tetrapods, our knowledge of the *Hox *genes and clusters has largely been restricted in several model organisms such as frogs, birds and mammals. Some evolutionary gaps still exist, especially for those groups with derived body morphology or occupying key positions on the tree of life, hindering our understanding of how *Hox *gene inventory varied along the sarcopterygian lineage.

**Results:**

We determined the *Hox *gene inventory for six sarcopterygian groups: lungfishes, caecilians, salamanders, snakes, turtles and crocodiles by comprehensive PCR survey and genome walking. Variable *Hox *genes in each of the six sarcopterygian group representatives, compared to the human *Hox *gene inventory, were further validated for their presence/absence by PCR survey in a number of related species representing a broad evolutionary coverage of the group. Turtles, crocodiles, birds and placental mammals possess the same 39 *Hox *genes. *HoxD12 *is absent in snakes, amphibians and probably lungfishes. *HoxB13 *is lost in frogs and caecilians. Lobe-finned fishes, amphibians and squamate reptiles possess *HoxC3*. *HoxC1 *is only present in caecilians and lobe-finned fishes. Similar to coelacanths, lungfishes also possess *HoxA14*, which is only found in lobe-finned fishes to date. Our *Hox *gene variation data favor the lungfish-tetrapod, turtle-archosaur and frog-salamander relationships and imply that the loss of *HoxD12 *is not directly related to digit reduction.

**Conclusions:**

Our newly determined *Hox *inventory data provide a more complete scenario for evolutionary dynamics of *Hox *genes along the sarcopterygian lineage. Limbless, worm-like caecilians and snakes possess similar *Hox *gene inventories to animals with less derived body morphology, suggesting changes to their body morphology are likely due to other modifications rather than changes to *Hox *gene numbers. Furthermore, our results provide basis for future sequencing of the entire *Hox *clusters of these animals.

## Background

The *Hox *genes are a large family of homeobox-containing transcription factors that control morphologies on the body axis of nearly all metazoans. Most of *Hox *genes normally consist of two exons with the conserved 180-bp homeobox located in exon2. In many animal species, *Hox *genes are arranged in genomic clusters with up to 15 distinct gene members [1] and, importantly, they are expressed in a "collinear fashion" -- anterior genes are expressed early in development and towards the front part of the embryo, posterior genes later in development and in more distal portions of the embryo [2]. Due to their important roles involved in patterning the anterior-posterior axis, modifications in *Hox *clusters might manifest in changes in the corresponding body regions; thus serve as a source of genetic innovations in shaping the diversification of metazoan body plans [3].

Because *Hox *genes are of particular interest in understanding the genetic basis of morphological evolution of metazoans, they are frequently among the first genes examined in an evolutionary context. Also *Hox *clusters have been characterized in a variety of animal species. Among chordates, the cephalochordate amphioxus possesses a single intact *Hox *gene cluster with 15 members [1]; in urochordate tunicates, the single cluster is secondarily broken and dispersed in the genome [4]. In contrast to these invertebrate chordates, primitive jawless vertebrates (lamprey and hagfish) possess three to seven *Hox *clusters, most probably through independent cluster duplications in the agnathan lineage [5-7]. Jawed vertebrates also have multiple clusters resulting from several rounds of genome-duplication events that occurred early in the evolution of vertebrates and some specific lineages. There are three or four clusters in chondrichthyans [8-10], four clusters in lobe-finned fishes [11-13] and tetrapods [14,15], up to eight in ray-finned fishes [16-20] and ~14 in tetraploid salmonid species [21]. The variations in vertebrate *Hox *clusters reflect a history of duplications and subsequent lineage-specific gene loss and can serve as models for studies of vertebrate genome evolution (reviewed by [14,22]).

For the sarcopterygian lineage (lobe-finned fishes plus tetrapods), all of its members investigated to date bear four clusters (*Hox*A, *Hox*B, *Hox*C, and *Hox*D) but the number of gene members varies among different groups. Mammals possess 39 *Hox *genes. For birds, a recent report on genomic annotation of *Hox *clusters in chicken [23] deduced that birds may have the same *Hox *gene inventory to mammals but two genes (*HoxC4 *and *HoxC5*) are still missing due to the incompleteness of the chicken genome. *In silico *survey of frog (*Xenopus tropicalis*) [14] and lizard (*Anolis carolinensis*) [15] revealed the persistence of *HoxC3 *(lost in mammals) in both species, but two genes (*HoxB13 *and *HoxD12*) are thought to have been lost in anuran amphibians. Recent relevant studies have further reported that the absence of *HoxB13 *in frogs also occurs in caecilians, and the loss of *HoxD12 *in frogs also happens in salamanders, caecilians [24] and snakes [25]. Most recently, the complete *Hox *clusters of the Indonesian coelacanth (*Latimeria menadoensis*), an early-branching sarcopterygian, have been sequenced [13]. Compared to the tetrapod lineage, the coelacanth possesses 42 *Hox *genes in total, lacking *HoxD13*, but retaining the four genes (*HoxC1*, *HoxC3*, *HoxB10*, and *HoxA14*) which were secondarily lost in mammals.

**Figure 1:**
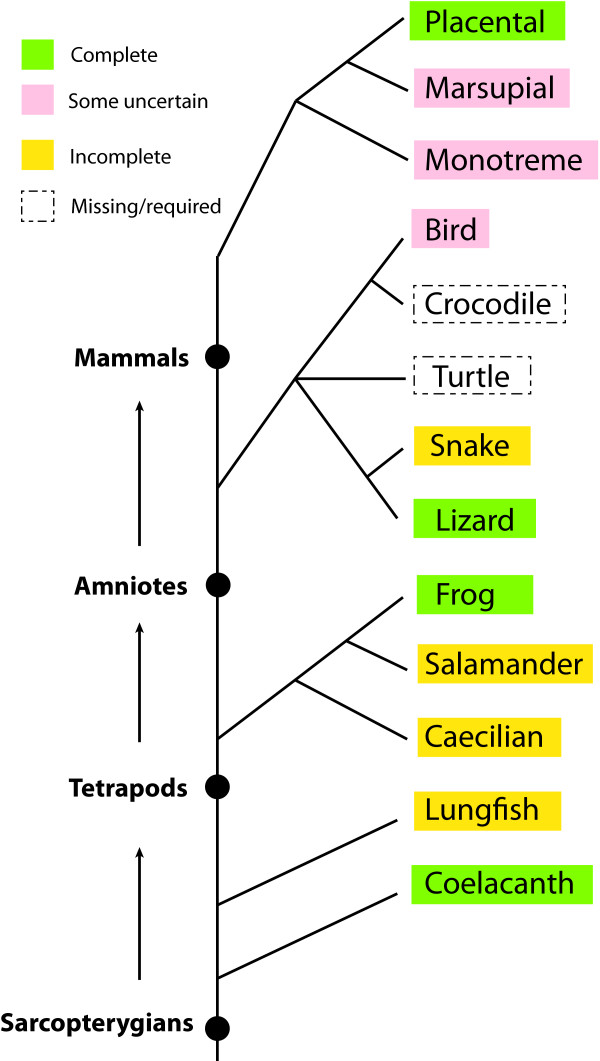
**Current status of the investigation of *Hox *gene inventory in different sarcopterygian groups along with a generally accepted tree for these groups**. We use a tritomy node for turtles, squamates (lizards and snakes) and archosaurs (birds and crocodiles), reflecting the current controversy on the relationships among them. Note that relevant information in most amphibian and reptile groups is either incomplete or missing.

However, our knowledge of the *Hox *genes and clusters along the sarcopterygian lineage remains incomplete because data from some major groups are still missing (illustrated in Figure 1). For example, it is curious to see whether lungfishes possess *Hox14 *and lost *HoxD13 *in their genomes (available data is rather incomplete, see [11]) as coelacanths. On the other hand, a "snake-like" morphology (limblessness and elongated body) repeatedly occurs in different groups of amphibians and reptiles, such as snakes, caecilians and some legless lizards. There were published reports suggesting the "snake-like" body morphology may be due to altered expression of *Hox *genes [25-27]. Comparative analyses on the *Hox *clusters among these special groups should be able to provide further hints on how such expression alterations happen. For sequencing of the *Hox *clusters, a framework investigation of *Hox *gene inventory in these groups (snakes, caecilians, et al.) is needed.

PCR surveys have demonstrated their value for preliminary identification of *Hox *genes in various animals [6,11,12,24]. However, PCR surveys of *Hox *genes often encounter a bias of the preference of degenerate primers and therefore, the actual number of existing genes is underestimated [6,24]. To circumvent this problem, we designed at least two sets of degenerated primers targeting a given *Hox *gene member, which increased the probability of successful amplification. Furthermore, when possible, we selected at least two species that span a broad evolutionary range for each tested groups, for which the primer preference may be different, maximizing the probability of finding all genes for a group. By adopting the two strategies, we carried out a comprehensive PCR survey for *Hox *genes in caecilians, salamanders, snakes, turtles, crocodiles, and lungfishes-the only other group of extant lobe-finned fishes beside coelacanths. We aimed to provide a more comprehensive understanding of *Hox *cluster evolution within the sarcopterygian lineage and present a general picture of *Hox *gene inventory variation among different sarcopterygian groups.

## Results and Discussion

### Amplification and Identification of *Hox *gene fragments

For each of the six sarcopterygian species examined, we tried different combinations of the degenerate primers, some targeted to several paralogue groups (PGs), some to specific groups and others to specific gene members, to amplify the homeobox-encoding region or exon1 of *Hox *genes (Table 1). We did several rounds of PCR first using "general" degenerate primers, and then using more specific primers designed for paralogue groups or gene members that were not found in the initial survey. A total of 3876 PCR fragments (80 to 165 bp long, depending on the primers used) were cloned and sequenced. Detailed information about the library construction and screening efficiency for the six sarcopterygian species and other relevant species is listed in the Additional file 1: Statistics of the sequenced clones. Occasionally, two sequences were found differing in only one to three nucleotides. When the nucleotide variations belong to synonymous substitutions and each of these sequences was present in more than one clone, they were considered as allelic variants. Conversely, non-synonymous substitutions of one or two nucleotides in only one clone but not the others of the same fragment were regarded as PCR or sequencing errors and excluded from further analyses.

**Table 1:** Primers used for amplification of *Hox *gene fragments

Target gene	Primer name	Sequence(5'-3')	AA sequences	PL* (bp)	Notes
**Forward primers (located at homeobox region)**					
PG1-PG7	HoxF1	TNGARYTNGARAARGARTTYCA	LELEKEFH	125	universal for PG1-PG7
PG1-PG10	HoxF1N1	CARACNYTNGARYTNGARAARGARTT	QTLELEKEF	128	universal for PG1-PG10
	HoxF1N2	CARGTNACNGARYTNGARAARGARTT	QLTELEKEF	128	universal for PG1-PG10
PG5-PG7	HoxF7S	CARACNTAYACNMGNTAYCARAC	QTYTRYQT	149	universal for PG5-PG7
PG8-PG10	HoxF8	TNGARAARGARTTYYTNTTYAA	LEKEFLFN	120	universal for PG8-PG10
	HoxF8N	TNGARYTNGARAARGARTTYYT	LELEKEFL	125	universal for PG8-PG10
PG9-PG11	HoxF9	DSNMGNAARAARVGNTGYCCNTA	TRKKRCPY	164	universal for PG9-PG11
PG1	HoxF1S	AAYTTYACNACNAARCARYTNAC	NFTTKQLT	149	universal for PG1
PG2	HoxF2S	MGGACNGCNTAYACNAAYACNCA	RTAYTNTQ	152	specific for PG2
PG3	HoxF3S	GCNTAYACNAGYGCNCARYTNGT	AYTSAQLV	149	specific for PG3
	HoxF3S1	MGVCCNMGVMGVBTNGARATGGC	RPRRVEMA	80	specific for PG3
PG4	HoxF4S	ACNGCNTAYACNMGNCARCARGT	TAYTRQQV	152	specific for PG4
PG5	HoxF5S	GGNAARMGSGCNMGSACNGC	GKRARTA	165	specific for A5, B5
	HoxFC5	AARCGNTCYMGAACNAGYTAYAC	KRSRTSYT	160	specific for C5
PG8	HoxF8S	GARAARGARTTYYTNTTYAAYCC	EKEFLFNP	116	specific for PG8
PG9	HoxF9S	GARAARGARTTYYTNTTYAAYATG	EKEFLFNM	116	specific for PG9
PG10	HoxF10S	AARMGNTGYCCNTAYACNAARCA	KRCPYTKH	152	specific for PG10
PG11	HoxF11	GARYTNGARMGNGARTTYTTYTT	ELEREFFF	122	specific for PG11
PG12	HoxF12	DSNMGNAARAARVGNAARCCNTA	SRKKRKPY	164	specific for PG12
	HoxF12C	AARCCNTAYTCNAARYTNCARAT	KPYSKLQL	149	specific for C12
	HoxF12D	AARCCNTAYACNAARCARCARAT	KPYTKQQI	149	specific for D12
	HoxF12N1	KCNMGVAARAARMGVAARCCSTA	SRKKRKPY	164	specific for PG12
	HoxF12N2	KCNMGVAARAARMGVAARCCWTA	SRKKRKPY	164	specific for PG12
	HoxF12N3	KCNMGVAARAARMGVAARACNTA	SRKKRKTY	164	specific for PG12
PG13	HoxF13A	GGNMGNAARAARMGNGTNCCNTA	GRKKRVPY	164	specific for PG13(A,C,D)
	HoxF13B	GGNMGNAARAARMGNATHCCNTA	GRKKRIPY	164	specific for PG13(B)
	HoxF13A1	CARYTRAARGARCTNGARMGNGARTA	QLKELEREY	128	specific for PG13(A)
	HoxF13B1	CARYTRAARGARCTNGARAANGARTA	QLKELENEY	128	specific for PG13(B,C,D)
**Reverse primers (located at homeobox region)**					
PG1-PG12	HoxR1	TTCATNCKNCKRTTYTGRAACCA	WFQNRRMK	--	universal for PG1-PG12
PG13	HoxR13	TTNACNCKNCKRTTYTGRAACCA	WFQNRRVK	--	specific for PG13
PG14	HoxR14	TCNGGNGTNAGRAANCGRTTYTC	ENRFLTPE	89	specific for PG14, used with HoxF13A/HoxF13B
					
**Primers used for amplification of exon1 of *Hox *genes**					
PG1	HoxB1(5'E1)F	GACATASTRYCNAAAGGTTGTAG	5' UTR	590-630	forward primer for HoxB1
	HoxB1(E1)R	TTAACYTTCATCCANTCRAANGT	TFDWMKVK	--	reverse primer for HoxB1
PG2	Hox2S(E1)F	GAATTYGAGMGRGARATHGGNTT	EFEREIGF	270-300	forward primer for PG2
	Hox2S(E1)R	YTTYTTCTCYTTCATCCANGG	PWMKEKK	--	reverse primer for PG2
PG3	Hox3S(E1)F	ATGCARAAARCRRCNTAYTAYGA	MQKATYYD	400-480	forward primer for PG3
	HoxC3(E1)F	ATGCAAAARGSTCYYTAYTAYGA	MQKGPYYE	400-480	forward primer for HoxC3
	HoxA3(E1)F	GCGACCTACTAYGAYAGYTCNGC	ATYYDSSA	390-470	forward primer for HoxA3
	HoxD3(E1)F	ATGCAGAAARCNGCYTAYTAYGA	MQKTAYYD	400-480	forward primer for HoxD3
	Hox3S(E1)R	TCYTTCATCCANGGRAADATNTG	QIFPWMKE	--	reverse primer for PG3
PG6	HoxB6(5'E1)F	AWACTRCTAATAGCTAAASCRCT	5' UTR	480-510	reverse primer for HoxB6
	Hox6S(E1)R	GARTTCATCCKYTGCATCCANGG	PWMQRMNS	--	reverse primer for PG6
PG7	HoxB7(5'E1)F	CTCGTAAAACCGACACTAAAACG	5' UTR	440-460	forward primer for HoxB7
	Hox7S(E1)R	CATCCARGGGTADATNCGRAA	FRIYPWM	--	reverse primer for PG7

* PL: Product length.

Initial BLAST searches in GenBank indicated that 82.9-96.5% sequenced clones belonged to *Hox *fragments, depending on the animal species examined. This result demonstrated the utility and efficiency of our newly designed *Hox *survey primers across most sarcopterygian lineages. Based on the phylogenetic analyses at the protein level, we can unambiguously assign 70-75% of the obtained homeobox sequences to exact *Hox *gene members. The phylogenetic signals in protein alignments of *Hox*2, *Hox*6-8 were especially weak so that the phylogenetic analyses at the nucleic acid level were performed to putatively distinguish *Hox *members for these paralogue groups. Using this strategy, we were able to determine the orthology of all obtained *Hox *fragments.

To test the credibility of our assignment of putative *Hox *fragments based on the nucleic acid phylogenetic analyses, we chose two species (*Naja atra *and *Ichthyophis bannanicus*) to perform the TAIL-PCR-based genome walking to get the 3' flanking sequences of their putative *Hox*6-8 fragments. The newly obtained sequences were compared with known sarcopterygian *Hox *genes, and all the assignments were verified.

### *Hox *gene inventories of different animal groups

#### Lungfishes

**Figure 2:**
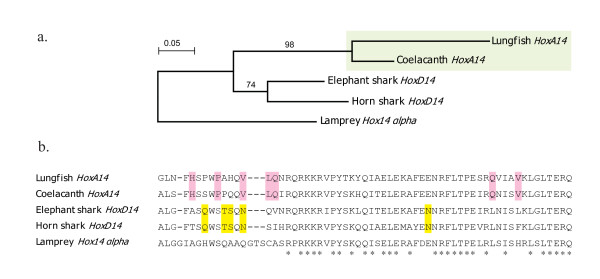
**Characterization of the exon2 of the lungfish *HoxA14 *gene**. (a) Neighbor-joining tree inferred from deduced protein sequences of the exon2 of *Hox14 *gene with JTT distance. Exon2 of *Hox14 *from lungfish (*Protopterus annectens*), coelacanth (*Latimeria menadoensis*), elephant shark (*Callorhinchus milii*) and horn shark (*Heterodontus francisci*) are included and the lamprey (*Lethenteron japonicum*) *Hox14 alpha *is used to root the tree. Bootstrap supports are given for each node above branches. (b) Protein alignment of *Hox14 *exon2 from the five species. Residues that are conserved in all species are indicated with asterisks below the alignment and that are diagnostic for *HoxA14 *or *HoxD14 *are highlighted with pink or yellow shading respectively.

*Protopterus annectens*, the African lungfish, was found to have a total of 42 *Hox *genes orthologous to genes of the four coelacanth *Hox *clusters. Generally the *Hox *inventory of the African lungfish is quite similar to that of the coelacanth (*Latimeria menadoensis*) [13], for example, they both possess *HoxC1*, *HoxC3 *and *HoxB10*; but interestingly the African lungfish lacks *HoxD12 *while the coelacanth has no *HoxD13*. Moreover, because the coelacanth has a *HoxA14 *gene and chondrichthyans have a *HoxD14 *gene [9,10] but all tetrapod species examined so far lack *Hox-14*, we wonder if *Hox14 *member also exist in lungfishes and/or the basal tetrapod lineage, i.e. caecilians. To this end, an 84-bp-fragment conserved only in *Hox14 *was tested by PCR in the African lungfish and the Banna caecilian and only the African lungfish gave the band with expected size, suggesting that *Hox14 *may have been lost in all tetrapods. However, no further assignment could be made for the lungfish *Hox14 *fragment because of its short size. We thus performed genome walking towards both ends of this *Hox14 *fragment and got full sequence of the exon2 of the lungfish *Hox14*. Using the lamprey *Hox14 alpha *as outgroup, phylogenetic analysis unambiguously assigned the lungfish's *Hox14 *member as a *HoxA14 *(Figure 2). Among all vertebrates investigated to date, only the coelacanths and the lungfishes which represent the only two groups of the extant lobe-finned fish possess *HoxA14*. Thus, *HoxA14 *seems to be characteristic of the lobe-finned fish *Hox *repertoire.

#### Caecilians

In the Banna caecilian (*Ichthyophis bannanicus*) 39 unique *Hox *gene fragments were found. Besides the core set of *Hox *genes (using mammals' as reference), we identified a *HoxC1 *fragment as well as a *HoxC3 *fragment in the Banna caecilian. The presence of *HoxC1 *in caecilians was further validated by screening the *Hox*1 library (see the Additional file 1: Statistics of the sequenced clones) in *Gymnopis multiplicata*, a representative of derived caecilians. Because *HoxC3 *was also found in the frog (*Xenopus tropicalis*; [15]) and the salamander (our survey, see below), it seems that all living amphibians retain *HoxC3*. Therefore we did not perform further survey for *HoxC3 *in other caecilian species. In addition, after having tried different combinations of primers, we could not find fragments of *HoxD12 *and *HoxB13 *in the three tested caecilian species (*I. bannanicus*, *G. multiplicata*, and *Typhlonectes natans*; see the Additional file 1: Statistics of the sequenced clones) which is consistent with the loss of *HoxD12 *and *HoxB13 *in caecilians previously reported by Mannaert *et al. *[24].

#### Salamanders

Compared with other animal groups, surveying the *Hox *gene inventory for salamanders was more difficult. We tried several species but in none of them could we find more than thirty-three *Hox *genes. In order to give a more integrated *Hox *gene inventory of salamanders, we combined the results from two species (*Batrachuperus tibetanus *and *Pachytriton brevipes*) to represent the group. Initial PCR survey of homeobox fragments in *Batrachuperus tibetanus*, the Tibetan mountain salamander, identified 33 *Hox *gene fragments after sequencing over 770 clones. Compared with the frog *Hox *complement, *HoxA1*, *HoxA3*, *HoxD3*, *HoxB6*, *HoxB7 *and *HoxA10 *were missing. The missing *Hox *genes were further surveyed in another salamander *Pachytriton brevipes *and two more *Hox *members (*HoxA1 *and *HoxA10*) were detected after sequencing 220 clones. To validate the potential presence of the remaining *Hox *members, we turned to detect their respective exon1s and finally fragments of *HoxA3*, *HoxD3*, *HoxB6 *and *HoxB7 *were found. Consistent with the previous reports of the presence of *HoxB13 *in salamandrids [24] and ambystomatids [Genbank: AF298184], we identified *HoxB13 *in the more basal hynobiid salamander (*B. tibetanus*), suggesting the presence of *HoxB13 *is likely a universal feature of all salamanders. Likewise, we did not detect *HoxD12 *in all salamanders investigated (see the Additional file 1: Statistics of the sequenced clones), in accordance with the previous survey result [24]. Altogether, salamanders have 39 *Hox *genes with the presence of *HoxC3 *and absence of *HoxD12 *and unlike the other two amphibian groups, salamanders possess *HoxB13*.

#### Frogs

The genomic architecture of *Hox *clusters of the frog (*Xenopus tropicalis*, a diploid frog species) has been previously reported [15] but *HoxB7 *was not detected in its genome due to a sequencing gap. However, *HoxB7 *mRNA was cloned in another frog (*Xenopus laevis*; accession: NM_001085641), indicating that frogs possess *HoxB7 *gene. In total, frogs have 38 *Hox *genes, lacking *HoxB13 *and *HoxD12*, but retaining *HoxC3*.

#### Lizards and Snakes

Di-Poï et al. [15] analyzed the genome data of the green anole lizard (*Anolis carolinensis*) and reported that lizards have 40 *Hox *gene with an additional *HoxC3 *gene which is absent in mammals. However, *HoxB13 *and *HoxD9 *were only deduced genes in their study, not directly detected due to some sequencing gaps. We reanalyzed the flanking sequences of these gaps and identified fragments orthologous to *HoxB13 *(exon2) and *HoxD9 *(exon1), improving the completeness of lizards' *Hox *gene inventory.

For snakes, we found 39 unique *Hox *sequences in the Chinese cobra (*Naja atra*). Like lizards, snakes also have a *HoxC3 *gene. To see if the presence of *HoxC3 *is a characteristic of all squamates, we further surveyed the *Hox*-3 genes in two other squamate species: gecko (*Hemidactylus bowringii*) and blind skink (*Dibamus bourreti*), belonging to two basal squamate groups Gekkonidae and Dibamidae, respectively. As a result, fragments of *HoxC3 *were unambiguously identified in the two species (see the Additional file 1: Statistics of the sequenced clones), suggesting all squamates should possess *HoxC3*.

Despite *HoxD12 *having been annotated in the green anole lizard, we did not detect *HoxD12 *fragment in the Chinese cobra after screening the *Hox*12 libraries constructed with different primer pairs. Absence of *HoxD12 *has just been reported in the corn snake recently [25]. Since both the Chinese cobra and the corn snake belong to derived snakes, to further test if *HoxD12 *is absent in all snakes, we surveyed *Hox*12 in two other snakes: blind snake (*Leptotyphlops blanfordii*) and ball python (*Python regius*) which occupy more basal positions on the Serpentes tree and might be able to circumvent the *Hox*12-specific primer bias if there were any. As a result, only *HoxC12 *fragment could be found in these two snakes as well (see the Additional file 1: Statistics of the sequenced clones). Therefore, it is likely that snakes have lost *HoxD12*.

#### Turtles

*Pelodiscus sinensis*, which is also known as the Chinese soft-shell turtle, was found to have 39 *Hox *genes, lacking *HoxC3 *compared with lizards. Since the squamate reptiles investigated so far have retained *HoxC3*, we wondered if the missing of *HoxC3 *in turtles was due to primer bias. So we surveyed *Hox*3 genes in another four turtles: the yellow-spotted Amazon river turtle (*Podocnemis unifilis*), the pig-nosed turtle (*Carettochelys insculpta*), the painted turtle (*Chrysemys picta*) and the red-eared slider turtle (*Trachemys scripta*), which were particularly selected to represent a broad evolutionary coverage for turtles. As a result, no fragments of *HoxC3 *could be identified (see the Additional file 1: Statistics of the sequenced clones), which is consistent with the loss of *HoxC3 *in turtles.

#### Crocodiles

For the Siamese crocodile (*Crocodylus siamensis*), most *Hox *genes were found as expected using the chicken *Hox *gene inventory [23] as reference. However, only one homeobox fragment was retrieved for *Hox*2 which usually contains two members (*HoxA2 *and *HoxB2*) though several primer combinations have been tried. Because the loss of *HoxA2 *or *HoxB2 *has never been reported in other vertebrates, we used primers targeting exon1 for *HoxA2 *and *HoxB2 *and were able to identify specific fragments for both genes in the Siamese crocodile. In addition, *HoxC3 *was not detected in the Siamese crocodile and its absence in crocodiles was further validated in the Chinese alligator (*Alligator sinensis*; see the Additional file 1: Statistics of the sequenced clones), a representative of the other major clade of living crocodiles. Thus crocodiles have 39 *Hox *genes and their *Hox *gene inventory is the same as that of mammals.

#### Birds

Though the genomic annotation of *Hox *clusters has been reported for the chicken (*Gallus gallus*), information for *HoxC4 *and *HoxC5 *is still missing due to incomplete genomic assembly [15,23]. And because of the gap at the 3' end of the *HoxC *cluster in the chicken genome, we do not know whether chicken has *HoxC3 *or not. Besides, we found the homeobox of the predicted chicken *HoxC12 *[Genbank: XM_426957] differs a lot from its orthologs in other vertebrates and may have assembly errors. In our PCR survey we confirmed the presence of chicken *HoxC4 *and *HoxC5 *by finding their respective homeobox fragments. To test whether birds retain *HoxC3*, we surveyed *Hox*3 genes in two birds: the domestic duck (*Anas platyrhynchos var. domestica*) and the ostrich (*Struthio camelus*), representing two major bird lineages (Neognathae and Paleognathae) respectively. As a result, *HoxC3 *was not detected, suggesting it was lost in birds (see the Additional file 1: Statistics of the sequenced clones). The reexamination of *Hox*12 in chicken, duck and ostrich confirmed the presence of *HoxD12 *in birds and revealed a fragment appearing well conserved with *HoxC12*s in other vertebrates, which, we think, represents the true bird *HoxC12 *(see the Additional file 1: Statistics of the sequenced clones). Hence, with the detection of *HoxC4*, *HoxC5*, *HoxC12 *and *HoxD12 *in birds, our data provides a more complete picture of avian *Hox *gene inventory.

#### Mammals

Although the *Hox *gene inventory for placental mammals (e.g., human and mouse) is clear, the relevant information for the other two major groups of extant mammals: marsupials, monotremes, has not been reported yet. We therefore performed an *in silico *survey of *Hox *genes for the grey short-tailed opossum *Monodelphis domestica *(version Broad/monDom5; URL: http://genome.ucsc.edu/cgi-bin/hgGateway?org=Opossum) and the duck-billed platypus *Ornithorhynchus anatinus *(version WUGSC 5.0.1/ornAna1; URL: http://genome.ucsc.edu/cgi-bin/hgGateway?org=Platypus). In both species, the *Hox *A, B, and D clusters are almost identical to those in human (*HoxB6 *was not detected in the platypus due to a sequencing gap). For the *HoxC *cluster, the platypus lacks genomic sequences covering *HoxC5*, *HoxC4 *and its 3' flanking region; the opossum lacks almost the entire genomic sequences of the *HoxC *cluster, except fragments of *HoxC6 *and *HoxC9*. Based on the observation that the architecture of *HoxC13-C4 *is conserved among all tetrapods, it is tempting to infer that marsupials and monotremes possess the same *HoxC *members as well. However, because the genomic sequences of the 3' flanking region of *HoxC4 *in both the opossum and the platypus are still missing, it would be premature to derive any conclusions about the presence/absence of *HoxC3 *in marsupials and monotremes.

#### The general scenario

**Figure 3:**
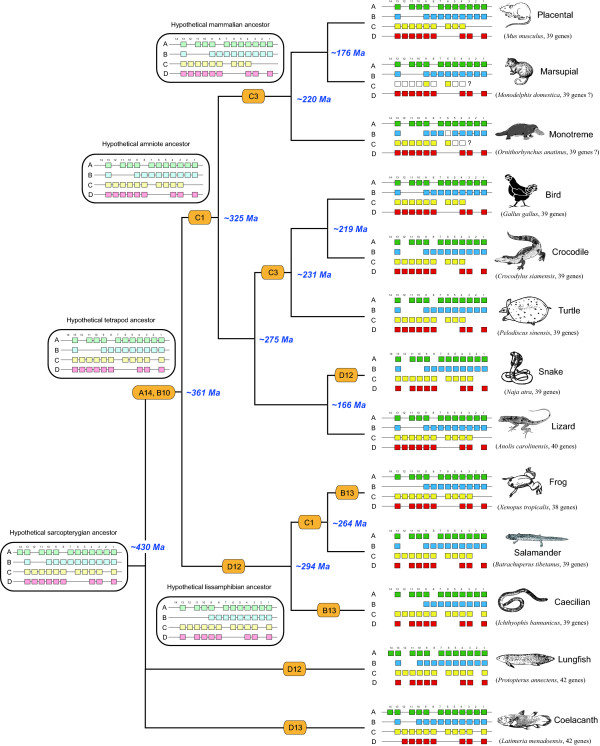
**Reconstructed evolutionary history of *Hox *cluster evolution within the sarcopterygian vertebrates**. Colored squares indicate *Hox *genes that have been identified; white squares are *Hox *genes that have not yet been sequenced but probably are present in the cluster(s). Solid lines connecting gene boxes indicate physical genomic linkage. Genomic sequences flanking *HoxC3 *gene in both opossum and platypus are still missing thus we consider the presence/absence of *HoxC3 *in marsupial and monotreme mammals is unknown yet (indicated by question marks). The gene inventory of the Hox clusters in the hypothetical ancestors of major evolutionary lineages are inferred based on parsimony principles, shown in open boxes above branches. Secondary losses of Hox genes are indicated in orange boxes along branches. A currently accepted phylogenetic tree is shown on the left, with divergence times (taken from [51]) shown beside nodes. Note that we tentatively favor a turtle-archosaur relationship based on *HoxC3 *variation among different amniote groups (see text for detailed discussion).

Combining our data with other published data, we are now able to provide a more complete scenario of how *Hox *gene inventory variation occurs along major sarcopterygian lineages, from lobe-finned fishes to mammals (Figure 3). On the whole, the four-cluster *Hox *architecture is well conserved; relatively basal lineages tend to retain more *Hox *gene members. The *HoxA14*, *B10*, *C1*, *C3 *were consecutively lost during the process of sarcopterygian evolution. *HoxD12 *and *HoxB13 *seem to be two hotspots of gene loss and different animal groups may have lost these *Hox *genes independently.

### *Hox *gene variation among sarcopterygian lineages and its evolutionary implications

#### The loss of HoxD12 is not directly related to digit reduction

Mannaert *et al. *[24] have proposed the absence of *HoxD12 *in amphibians be related to the absence of the fifth finger as frogs and salamanders normally have only four fingers and caecilian is limbless. In such a view, *HoxD12 *would be frequently lost in limbless animals (no digits at all). The hypothesis seems reasonable because *HoxD12 *does be lost in the snake (limbless, no digits) and the African lungfish (only with thread-like fins) according to our *Hox *gene survey. However, besides snakes, there are many other squamates with snake-like, limbless body forms. It is necessary to test the hypothesis more strictly in these limbless animals as well. To this end, we further surveyed *Hox*12 genes in other limbless lizards, such as *Amphisbaena caeca *(Amphisbaenidae), *Blanus strauchi *(Blanidae), *Ophisaurus harti *(Anguidae), *Anniella pulchra *(Anniellidae), *Typhlosaurus sp. *(Scincidae) and *Dibamus bourreti *(Dibamidae), representing a broad evolutionary coverage for squamates. To our surprise, both *HoxC12 *and *D12 *could be unambiguously detected in all these limbless animals (see the Additional file 1: Statistics of the sequenced clones). This result indicated that the loss of *HoxD12 *is not as directly related to the digit-reduction phenotype as previously proposed.

#### The significance of HoxD13 retention in lungfishes

While the other group of extant lobe-finned fish, the coelacanths, has lost *HoxD13 *[13], a *HoxD13 *fragment was identified from the African lungfish in our PCR survey. Previous knock-out experiments in mice have demonstrated that *HoxD13 *is essential in the autopodium formation for tetrapods [28,29], so it is easy to understand the fact that all tetrapods investigated so far possess the *HoxD13 *gene. Therefore, the retention of *HoxD13 *which is shared by tetrapods and lungfishes but not coelacanths, is consistent with lungfishes being the closest living sistergroup of tetrapods, a widely accepted relationship among coelacanths, lungfishes and tetrapods [30].

**Figure 4:**
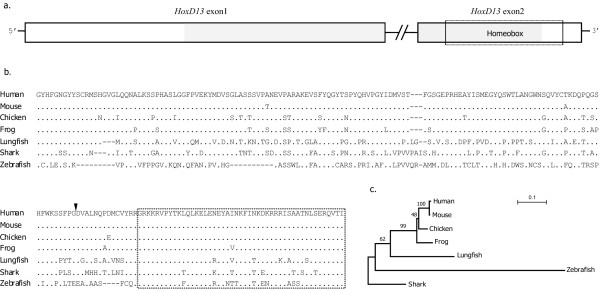
**Comparison of vertebrate HoxD13 protein sequences**. (a) Diagram of the structure of *HoxD13 *gene. The *HoxD13 *gene comprises two exons (shown as open boxes), and the homeobox region locates in the exon2 (indicated by dash line frame). (b) Partial protein alignment of *HoxD13 *(shaded region in Figure 4a) from human (*Homo sapiens*), mouse (*Mus musculus*), chicken (*Gallus gallus*), frog (*Xenopus tropicalis*), lungfish (*Protopterus annectens*), elephant shark (*Callorhinchus milii*) and zebrafish (*Danio rerio*). Exon-intron splicing site is indicated as black triangle and the homeobox region is shown in a dash line frame. (c) Neighbor-joining tree (JTT distance) inferred from the above protein alignment with bootstrap supports of 1,000 iterations.

Strictly, small *Hox *gene fragments generated by PCR surveys are not definite evidences for the absence/presence of a gene, for example, the *HoxD13 *fragment in the goldfish *Carassius auratus auratus *is thought to represent a pseudo gene [31]. Therefore, we wonder whether the African lungfish *HoxD13 *is really a functional gene. To address this issue, genome walking was performed to obtain the 5' region of the homeobox of the lungfish *HoxD13*, including the intron and the complete exon1. No premature stop codons were observed, indicating the African lungfish *HoxD13 *is not a pseudo gene. Sequence alignments and phylogenetic analysis show that, the tetrapod *HoxD13s *are more similar to the African lungfish *HoxD13 *than to that of bony fishes (zebrafish) or cartilaginous fishes (shark) (Figure 4). Although the lungfish has no "digits", its *HoxD13 *may represent the intermediate form towards the functions in tetrapod *HoxD13*s during the fin-to-limb transitional history. In the future it would be interesting to perform "replacement" experiments in mice to see to what extent the lungfish *HoxD13 *could rescue the functions of the tetrapod *HoxD13 *especially in terms of autopodium formation.

#### The variation of HoxC3 shows clues for turtles' position on the amniote tree

The phylogenetic position of turtles is the most controversial issue in the reconstruction of the living amniote tree of life. After many different kinds of investigations from both molecular and morphological data, four main hypotheses concerning the phylogenetic relationships of turtles to the other groups of living amniotes have been proposed [30]: (*A*) Turtles as the only living representatives of anapsid reptiles, and as the sister-group of diapsid reptiles, i.e., the Lepidosauria (the tuatara, snakes, and lizards) + Archosauria (crocodiles and birds); (*B*) Turtles placed within diapsids, and as the sister-group of the Lepidosauria; (*C*) Turtles as diapsids, and as the sister-group of the Archosauria; (*D*) Turtles as diapsids, but placed inside the Archosauria, and as the sister-group of crocodiles. Most morphological studies favor either Hypothesis *A *[32-35] or *B *[36,37] and Hypothesis *A *is the traditional view of the placement of turtles. In contrast to morphological views, recent molecular phylogenetic studies tend to support either Hypotheses *C *or *D *and reject Hypotheses *A *and *B *[38-43]. However, due to a severe slow down of substitution rate in turtles relative to diapsid reptiles [43], we can not rule out the possibility that the molecular turtle-archosaur relationship is caused by analytical artifacts. Therefore, besides traditional morphological inferences and sequence-based molecular phylogenetic analyses, a third form of data is needed to explore and test the alternative phylogenetic hypotheses of the turtle's placement.

The presence of *HoxC3 *gene among living amniote lineages seems a good indicator of their interrelationships. Since both lobe-finned fishes and amphibians possess *HoxC3*, the presence of *HoxC3 *is most likely the ancestral state for amniotes. According to our survey, for living amniotes, only squamates (snakes and lizards) retained *HoxC3 *(the tuatara data is missing here, but it will not alter our inference due to its affinity to squamates) and other groups (mammals, birds, crocodiles and turtles) all lost this gene. Following the principle of Dollo parsimony - which assumes that losses of genes are much more common and likely than independent evolutionary origins, - we can evaluate the four hypotheses about the position of turtles mentioned above. Both Hypotheses *A *and *B *require 3 steps of independent loss of *HoxC3 *in mammals, turtles and archosaurs while Hypotheses *C *and *D *need only two steps. Consequently, our *Hox *gene inventory data is in line with most recent molecular studies favoring a turtle-archosaur relationship but unable to discriminate between Hypotheses *C *and *D*. Considering that Archosauria is a well supported clade, we tentatively accept Hypothesis *C *and illustrate turtles' position as shown in Figure 3.

#### The retention of HoxC1 in caecilians supports the Batrachia hypothesis

Because sharks, many teleost fishes, lobe-finned fishes all possess *HoxC1 *but all tetrapod species examined before this study lack this gene, Kuraku and Meyer [22] deduced that tetrapod ancestors lost their *HoxC1 *gene when they diverged from lobe-finned fishes. However, our finding of *HoxC1 *in caecilians suggested that tetrapod ancestors actually retained *HoxC1 *gene but subsequently lost in different lineages. The presence of *HoxC1 *likely represents a "fish-style" *Hox *gene inventory and only basal tetrapod lineages have the possibility to retain this gene. Amphibians definitely branch first from the tetrapod tree and comprise of three distinct living groups: frogs, salamanders and caecilians [44]. The retention of *HoxC1 *in caecilians but not in frogs and salamanders implied that among the three living amphibian groups, caecilians are more distantly related to frogs and salamanders, supporting the Batrachia hypothesis (a frog+salamander clade) advocated by most recent molecular studies [45-48].

## Conclusions

We performed a comprehensive PCR survey of *Hox *genes for six major sarcopterygian groups: lungfishes, caecilians, salamanders, snakes, turtles and crocodiles and clarified some uncertainties of birds' *Hox *gene inventory. Our study provided a more complete scenario for evolutionary dynamics of *Hox *genes along major sarcopterygian lineages. On the whole, *Hox *gene inventories of sarcopterygians are rather conserved with only little variations occurring in the anterior or posterior *Hox *paralogue groups. The *Hox *gene inventories of limbless caecilians and snakes largely resemble those of animals with less derived body morphology, suggesting changes to their body morphology were likely due to other modifications rather than changes to *Hox *gene numbers. In future, it is interesting to sequence the entire *Hox *clusters for these animals and our work can serve as basis for this purpose.

## Methods

### Taxon sampling

In order to obtain a broad overview of *Hox *gene variation in major sarcopterygian lineages, we focused on those groups whose *Hox *gene data were incomplete or missing when our research began. The following six species were selected for comprehensive *Hox *gene survey: *Protopterus annectens *(African lungfish) which represents another major group of extant lobe-finned fishes besides coelacanths; *Ichthyophis bannanicus *(Banna caecilian) and *Batrachuperus tibetanus *(Tibetan mountain salamander) representing caecilians and salamanders for the amphibian lineage; *Naja atra *(Chinese cobra), *Pelodiscus sinensis *(Chinese soft-shell turtle) and *Crocodylus siamensis *(Siamese crocodile) as the representative of snakes, turtles and crocodiles for the reptilian lineage, respectively. As we found some *Hox *gene variations among these group representatives, we selected additional 22 species, providing a broad evolutionary coverage for different animal groups, to test the observed *Hox *variation within certain group. Detailed information for all species used in this study is listed in Table 2.

**Table 2:** List of species used in this study

Taxonomy		Scientific name	Common name	Collection locality (or specimen voucher No.)
**Species used for PCR survey**				
Sarcopterygii	Dipnoi	*Protopterus annectens*	African lungfish	Pet trade
Amphibia	Gymnophiona	Ichthyophis bannanicus*	Banna caecilian	Beiliu, Guangxi, China
	Caudata	*Batrachuperus tibetanus*	Tibetan mountain salamander	Qingchuan, Sichuan, China
Reptilia	Serpentes	Naja atra*	Chinese cobra	Shaoguan, Guangdong, China
	Testudines	*Pelodiscus sinensis*	Chinese softshell turtle	Shaoguan, Guangdong, China
	Crocodylia	*Crocodylus siamensis*	Siamese crocodile	Shenzhen, Guangdong, China
**Species used for *Hox *gene member validation**				
Gymnophiona	Caeciliidae	Gymnopis multiplicata*	Purple caecilian	MVZ Herps 228795
	Typhlonectidae	Typhlonectes natans*	Rubber eel	MVZ Herps 179733
Caudata	Salamandridae	*Pachytriton brevipes*	Chinese fat newt	Anji, Zhejiang, China
	Hynobiidae	*Batrachuperus yenyuanensis*	Yenyuan stream salamander	Xichang, Sichuan, China
Serpentes	Pythonidae	Python regius*	Ball python	Personal captivity
	Leptotyphlopidae	Leptotyphlops blanfordii*	Blind snake	MVZ Herps 236621
Squamata	Dibamidae	Dibamus bourreti*	Bourret's blind skink	Hongkong, China
	Gekkonidae	*Hemidactylus bowringii*	House Gecko	Guangzhou, Guangdong, China
	Anguidae	Ophisaurus harti*	Hart's glass lizard	Pet trade
	Scincidae	Typhlosaurus sp. *	Legless skink	MVZ Herps 164850
	Blanidae	Blanus strauchi*	Anatolian worm lizard	MVZ Herps 230227
	Amphisbaenidae	Amphisbaena caeca*	Puerto Rican worm lizard	MVZ Herps 232753
	Anniellidae	Anniella pulchra*	California legless lizard	MVZ Herps 230670
	Bipedidae	*Bipes biporus*	Baja worm lizard	MVZ Herps 236257
Testudines	Podocnemididae	*Podocnemis unifilis*	Yellow-spotted Amazon river turtle	Pet trade
	Carettochelyidae	*Carettochelys insculpta*	Pig-nosed turtle	Pet trade
	Emydidae	*Chrysemys picta*	Painted turtle	MVZ Herps 241506
		*Trachemys scripta*	Red-eared slider turtle	Commercial food source
Crocodylia	Alligatoridae	*Alligator sinensis*	Chinese alligator	Alligator Research Center, Xuanzhou, Anhui, China
Aves	Paleognathae	*Struthio camelus*	Ostrich	Commercial food source
	Galliformes	*Gallus gallus domesticus*	Chicken	Commercial food source
	Anseriformes	*Anas platyrhynchos var. domestica*	Domestic duck	Commercial food source

* Species that is limbless or only has limb remnants

### PCR, cloning and sequencing of *Hox *genes

We amplified fragments of *Hox *genes from genomic DNA by PCR using several combinations of degenerate primers flanking the homeobox or exon1 region (Table 1). In the comprehensive surveys for the group representatives, *Hox *genes were first divided into six paralogue group (PG) sets (PG1-7, PG1-10, PG11, PG12, PG13, PG14) for amplification. For some paralogue groups such as PG12 and PG13 which were difficult to amplify, more than one set of primers was used to increase the probability of successful amplification. If any members of PG1-10 was not initially retrieved with the general primers, e.g., *HoxC5 *of PG5 could not been found, a PG specific forward primer HoxF5S would be used; if the PG specific primers still failed to amplify the gene, a more specific primer HoxFC5 would be used to confirm its presence or absence. PG specific primers were also applied in the subsequent confirmation of *Hox *gene variations in the additional 22 species. We know that non-detection by PCR survey can not be interpreted definitively as a missing gene, but by trying more sets of primers and surveying more numbers of related species, the completeness of *Hox *gene PCR survey for a given animal group is expected to be high.

PCR was performed in 25 μl reaction volumes with ExTaq DNA polymerase (Takara, Dalian) using the following cycling parameters: an initial denaturation step at 94°C for 4min, 45 cycles of 94°C for 30s, 42-55°C for 1min, 72°C for 30s, and a final extension step at 72°C for 10min. PCR products were purified by agarose gel extraction (Axygen, Suzhou) and cloned into a PMD19-T vector (Takara, Dalian). Positive recombinant clones were identified by colony PCR and the PCR products were cleaned with ExoSap treatment and sequenced on an automated ABI3730 DNA sequencer.

### Sequence analysis

Firstly, an alignment of homeobox regions of *Hox *genes from six well-studied vertebrate species was made. *Hox *genes of coelacanth (*Latimeria menadoensis*), frog (*Xenopus tropicalis*), chicken (*Gallus gallus*), lizard (*Anolis carolinensis*), mouse (*Mus musculus*) and human (*Homo sapiens*) were retrieved from GenBank upon availability. For some *Hox *genes of frog, lizard and chicken that can not be directly collected from GenBank, we identified their draft sequences by alignments with other vertebrates at the UCSC Genome Browser http://genome.ucsc.edu/. All the obtained sequences were cut down to 180 bp of homeobox and aligned by ClustalX [49].

For each of the examined species, we compared all its obtained sequences against each other and identified a set of unique sequences for the species. These unique sequences were first screened for *Hox *gene fragments using BLAST searches in GenBank. Candidate sequences were then aligned to the aforementioned 6-species homeobox alignment, both at the protein and the nucleic acid level. Phylogenetic trees were generated by the Neighbor-Joining method implemented in the MEGA 4.0 [50] with either K2P (for nucleic acid) or JTT (for protein) distances. Supports for the nodes were evaluated by non-parametric bootstrap analyses of 1,000 replicates. The assignment of the candidate sequences were made based on their phylogenetic position at the protein or the nucleic acid level.

### Genome walking

Because the homeobox regions we used are relatively short, some *Hox *gene fragments cannot be undoubtedly assigned to certain paralogue. To confirm the credibility of our assignments, we performed genome walking (GW) to obtain the unknown sequence adjacent to the homeobox region towards 5' or 3' end of exon2. The homeobox-flanking regions are less conserved and more informative and thus can facilitate identification of the paralogue. The genome walking was carried out by using the Genome Walking Kit (Takara, Dalian) which is based on a TAIL-PCR technique. GW-specific primers were designed based on the candidate fragment sequences following the manufacture's guidance (available upon request).

### Sequence availability

All sequences of *Hox *gene fragments identified in this paper are deposited in GenBank under accession numbers HQ441256 to HQ441561

## Authors' contributions

DL and PZ conceived the study. PZ designed the experiments. RW and JG carried out the PCR survey and the genome walking experiments. DL, PZ and RW analyzed the data. CW provided the alligator sample. DL, PZ and RW wrote the manuscript. All authors read and approved the final manuscript.

## Supplementary Material

Additional file 1 **Statistics of clones sequenced in each species and during the validation of *HoxC1, HoxC3, HoxD12 and HoxB13***. Click here for file
